# Vertical root fracture detection with cone-beam computed tomography in Biodentine™ filled teeth

**DOI:** 10.1186/s12903-024-04947-7

**Published:** 2024-10-04

**Authors:** Jakob W. G. Van Acker, Charlotte Yvergneaux, Wolfgang Jacquet, Melissa Dierens, Geert Hommez, Joris Van Acker, Matthieu Boone, Sivaprakash Rajasekharan, Luc C. Martens

**Affiliations:** 1 ELOHA (Equal Lifelong Oral Health for All) research group, Paediatric Dentistry, Oral Health Sciences, Ghent University, Ghent, Belgium; 2 Localities Ontologies Commons Integrated (LOCI), Vrije Universiteit Brussel (VUB), Brussels, Belgium; 3 Oral Health Research Group (ORHE), Vrije Universiteit Brussel (VUB), Brussels, Belgium; 4 Department of Periodontology and Oral Implantology, Ghent University, Ghent, Belgium; 5 Oral Health Sciences, Faculty of Medicine and Health Sciences, Ghent University, Ghent, 9000 Belgium; 6 UGent-Woodlab - Laboratory of Wood Technology, Department of Environment, Ghent University, Coupure links 653, Ghent, 9000 Belgium; 7 Centre for X-ray Tomography, Physics and Astronomy, Ghent University, Ghent, Belgium

**Keywords:** Cone-beam computed tomography, Tooth fractures, Diagnosis, Endodontics, Radiography

## Abstract

**Purpose:**

This study aimed to evaluate the accuracy of detecting vertical root fractures in Biodentine™-filled teeth using the Promax 3Dmax cone-beam computed tomography (CBCT) unit compared to periapical radiographs. It tested hypotheses regarding CBCT’s diagnostic superiority in non-root-filled and Biodentine™-root-filled maxillary central incisors and assessed the impact of smaller field of view and lower intensity settings on detection accuracy.

**Materials and methods:**

Extracted maxillary incisors were divided into groups based on fracture status and root filling material, then placed in a Thiel-embalmed skull to simulate clinical conditions. The teeth were imaged using periapical radiographs and the CBCT unit under different settings. Fracture thickness was measured with microcomputed tomography for accuracy benchmarking. Multiple observers assessed the images, and statistical analyses were conducted to evaluate diagnostic performance.

**Results:**

Intra-rater reliabilities of consensus scores ranged from good to very good. Specificities were generally higher than sensitivities across all imaging modalities, but sensitivities remained constantly low. None of the Area Under the Curve scores exceeded 0.6, indicating poor overall accuracy for all imaging modalities. Paired comparisons of the area differences under Receiver Operator Characteristic curves revealed no significant differences between the CBCT and periapical radiograph techniques for detecting vertical root fractures in either Biodentine™-filled or non-root-filled teeth.

**Conclusions:**

There was no significant accuracy improvement of the current CBCT device (Promax 3Dmax, Planmeca, Finland) over periapical radiographs in detecting small vertical root fractures in both non-root-filled and Biodentine™-root-filled maxillary central incisors. A smaller field of view with lower intensity did not enhance detection accuracy. These results highlight the challenges in accurately detecting small VRFs, emphasizing the need for further research and technological advancements in this domain.

**Supplementary Information:**

The online version contains supplementary material available at 10.1186/s12903-024-04947-7.

## Introduction

Longitudinal tooth fractures encompass a spectrum of pathologies, including craze lines, fractured cusps, cracked teeth, split teeth, and vertical root fractures (VRF) [1]. A VRF, characterized by a complete or incomplete fracture initiated from the root at any level, primarily oriented facio-lingually, poses a particular diagnostic challenge for dentists [1]. This challenge is exacerbated by the increased prevalence of VRFs in endodontically treated teeth [2, 3]. While most cases manifest in premolars and molars, some studies report 9–17% of detected VRFs to be present in maxillary central incisors [3, 4].

The diagnosis of VRFs remains intricate, as clinical symptoms and indirect radiographic signs are far from pathognomonic. Timely and definitive diagnosis is crucial in preventing extensive damage to surrounding tissues and averting unnecessary treatments [2].

While conventional periapical radiographs (PRs) are readily available in dental practices, their sensitivity in VRF detection is limited [5, 6]. In contrast, Cone-Beam Computed Tomography (CBCT) offers a three-dimensional image without superimposition, enlargement, and deformation, although this advantage comes with an increased radiation exposure [7]. Additionally, CBCT entails a higher and variable monetary cost compared to conventional imaging methods [8, 9]. It is also important to note that highly radiopaque materials, commonly found in dental restorations, can introduce artefacts that significantly compromise the diagnostic quality of the image [10]. Despite these considerations, there is scarcity of studies evaluating the diagnostic accuracy of PRs and CBCT for detecting smaller incomplete VRFs [11–14].

Existing research primarily delves into the effects of gutta-percha and various posts and occasionally different sealers on VRF detection using CBCT and PRs [15, 16]. Notably, studies such as those by Bahmani et al., 2021 and Cavalcanti et al., 2022 have examined the influence of low radiopaque bioceramic sealers combined with gutta-percha on VRF detection [17, 18]. However, these studies do not specifically address the impact of full root restorations using other endodontic materials, such as Biodentine™ (Septodont, France), which is characterized by its lower radiopacity compared to gutta-percha. Biodentine™, a dental material composed primarily of tricalcium silicate, dicalcium silicate, calcium carbonate, and zirconium oxide, shares compositional similarities with natural tooth minerals, rendering it biocompatible and versatile in dental procedures. Its applications have expanded over the years, and it is now increasingly employed for a range of endodontic purposes, including the management of deep caries, exposed pulp, resorption lesions, treatment of immature roots after trauma, and addressing root fractures [19–22]. Notable advantages include its biocompatibility, rapid setting time, effective sealing properties, and the ability to stimulate hard-tissue formation. In contrast to gutta-percha, Biodentine™ exhibits lower radiopacity [19, 20, 23].

This study seeks to address an essential gap in the literature by comparing the in vitro accuracy of PRs and CBCT in detecting small VRFs (thickness < 200 μm) in maxillary central incisors. The investigation further explores the impact of different factory-predetermined CBCT settings, tailored for both adults and children, in non-root-filled and Biodentine™-root-filled maxillary central incisors.

The null hypotheses are as follows:

- H0^1^ - CBCT exhibits significantly higher accuracy in detecting small VRFs compared to PRs in non-root-filled maxillary central incisors in vitro.
- H0^2^ - CBCT exhibits significantly higher accuracy in detecting small VRFs compared to PRs in Biodentine™-root-filled maxillary central incisors in vitro.
- H0^3^ - CBCT with a smaller field of view (FOV) but lower intensity exhibits significantly higher accuracy in detecting small VRFs in maxillary central incisors in vitro.

## Materials and methods

**Fig. 1:**
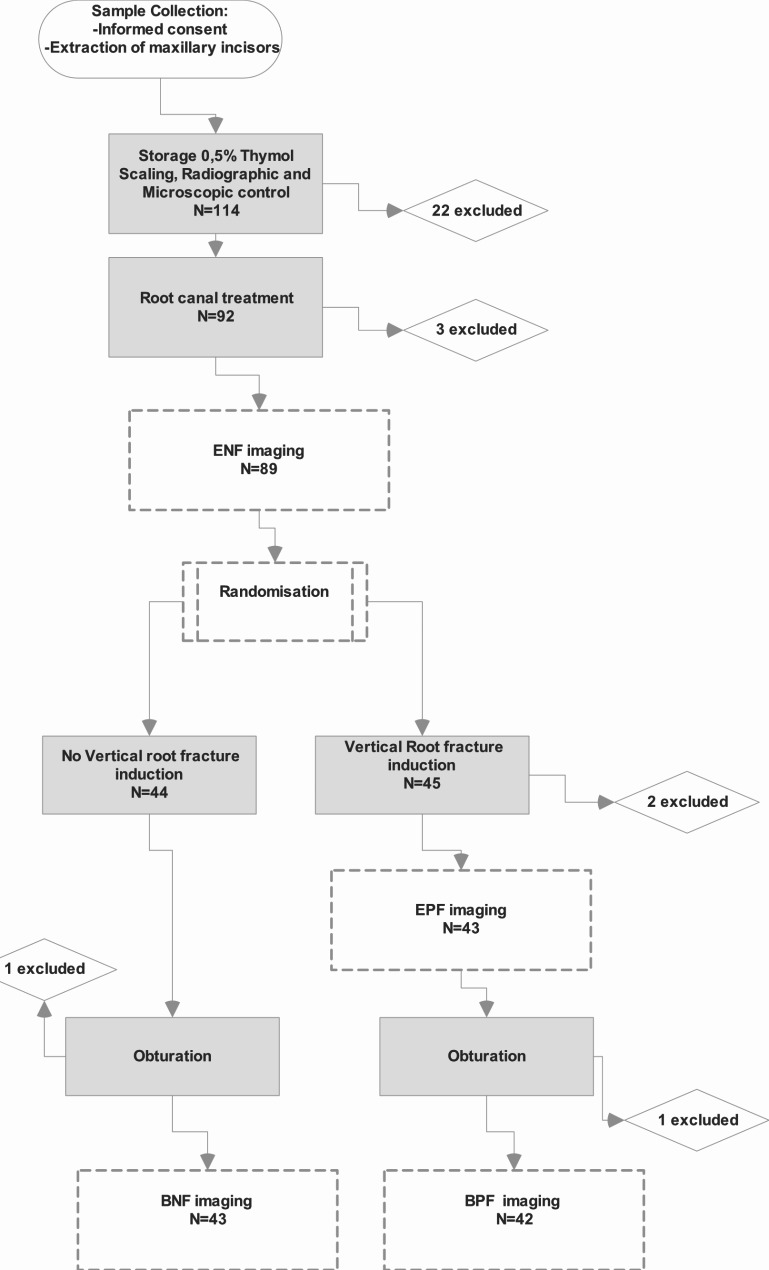
A flow diagram providing a visual overview of the included teeth, examinations, treatments and exclusions; ENF, Empty root canal- No Fracture; EPF, Empty root canal- Partial Fracture; BNF, Biodentine™ filled root canal- No Fracture; BPF, Biodentine™ filled root canal- Partial Fracture

A flow diagram providing a visual overview of the included teeth, examinations, treatments and exclusions can be appreciated in Fig. 1.

### Sample collection

This study was accepted by the ethical committee of the Ghent University hospital (BC16/1399–1400). The authors retrieved informed consents for using the teeth from patients who received an extraction of a maxillary central incisor.

### Sample preparation

**Fig. 2:**
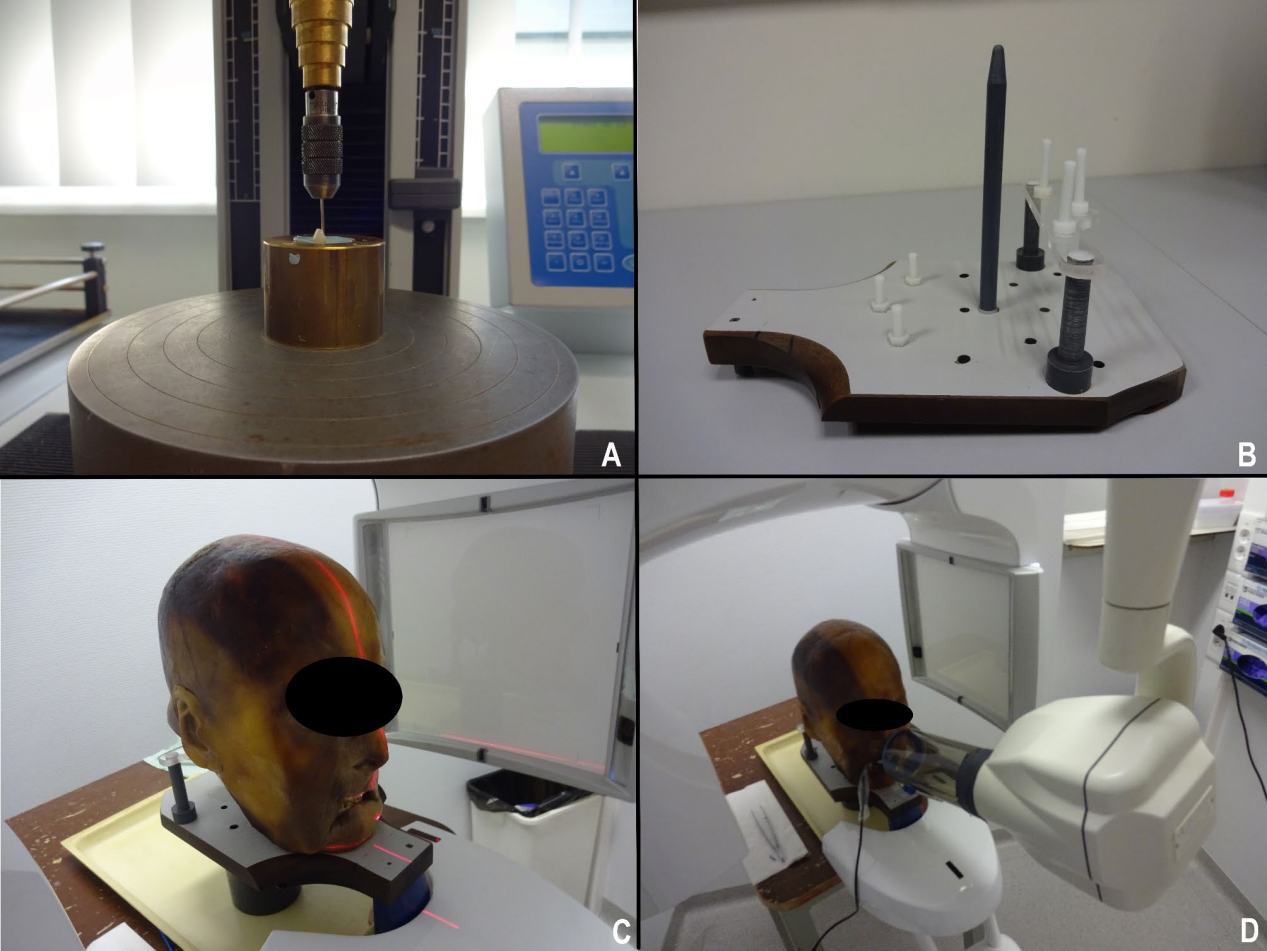
Material and methods: (**a**) Fracture initiation. (**b**) Custom platform for skull fixation. (**c**) CBCT imaging of the samples. (**d**) Periapical imaging of the samples

Images of the positioning and imaging of samples can be appreciated in Fig. 2.

All maxillary incisors were stored in 0.5% thymol. Two researchers (JVA and CY) checked the samples radiographically and microscopically for the absence of root canal filling materials, fractures and root resorptions. Radiographs were taken using a dental X-Ray device (Satelec X-Mind^®^, Satelec, France) and a digital phosphor plate system (Vistascan Perio Plus^®^, Dürr Dental Benelux, Belgium) with the following settings: 65 kV, 7 mA, 0.16s. Two radiographs were taken, one in a mesiodistal and one in a buccolingual direction. The microscope used was a Novex K-Range (Novex, USA) with a 40x maximum magnification.

The root canals were prepared with rotary instruments (Protaper, Dentsply Sirona, USA) until size F5. Further preparation was performed with hand instruments (K-files, Dentsply Sirona, USA) until a passive apical fit with a master apical file 70 was reached. During the preparation of the root canals and after shaping with rotary and hand instruments, the canals were thoroughly rinsed with a 2.5% sodium hypochlorite solution to disinfect and remove debris. Following the sodium hypochlorite rinse, the canals were flushed with a saline solution to neutralize any residual hypochlorite. Finally, the canals were dried using sterile paper points (Maxima^®^ paper points, Henry Shein, USA). After this, the same researchers performed the radiographic imaging of this control group ENF (Empty root canal- No Fracture, *n* = 89).

Partial VRFs were induced in 45 teeth selected randomly using a random sequence generator (random.org). Root fractures (Fig. 2a) were induced according to Brady et al., 2014 [11]. The sewing needle was a Prym short darner size 9, and the tensile testing machine an LRX-plus (Ametek company, USA). The operator (JVA) inspected the sample for an incomplete root fracture, defined as a (microscopically) visible longitudinally oriented crack within the root without complete separation of the root fragments. The microscope used was a Novex K-Range (Novex, USA) with a 40x maximum magnification. Two teeth were excluded, one because the tooth was utterly fractured (*n* = 1) and one because a fracture could not be induced (*n* = 1). Forty-three teeth with VRF were thus included in the study. The radiographic imaging step was repeated for the group EPF (Empty root canal- Partial Fracture, *n* = 43).

Before placement of the Biodentine™, the canals were flushed with a saline solution and dried again using sterile paper points. Biodentine™ was prepared according to the manufacturer’s instructions. This involves mixing the powder and liquid components to achieve a homogenous, workable paste. The mixed Biodentine™ was then introduced into the prepared root canal. This was done using lentulo spirals (Endoflex Paste Fillers − 21 mm, 1–4 (ISO 025–040), Henry Shei, USA) and an endodontic obturator (Black Line − 9/11, Hu FriedyGroup, USA) to ensure thorough and even distribution of the material throughout the canal. Care was taken to avoid voids or gaps within the filling. A total of 44 teeth without root fracture and 43 teeth with root fracture were obturated with Biodentine™. One tooth per group was lost in this process. The radiographic imaging step was then repeated for the group BNF (Biodentine™ filled root canal- No Fracture, *n* = 43) and BPF (Biodentine™ filled root canal- Partial Fracture, *n* = 42).

### Sample positioning

All teeth were positioned in the maxilla of a dentate Thiel embalmed skull for hard and soft tissue-mimicking [24]. A custom Plexiglas platform (Fig. 2b) was attached to the CBCT device (Promax 3Dmax, Planmeca, Finland) on which the Thiel embalmed skull was fixated with multiple acryl pins for reproducibility.

### X-ray imaging: PR and CBCT

**Fig. 3:**
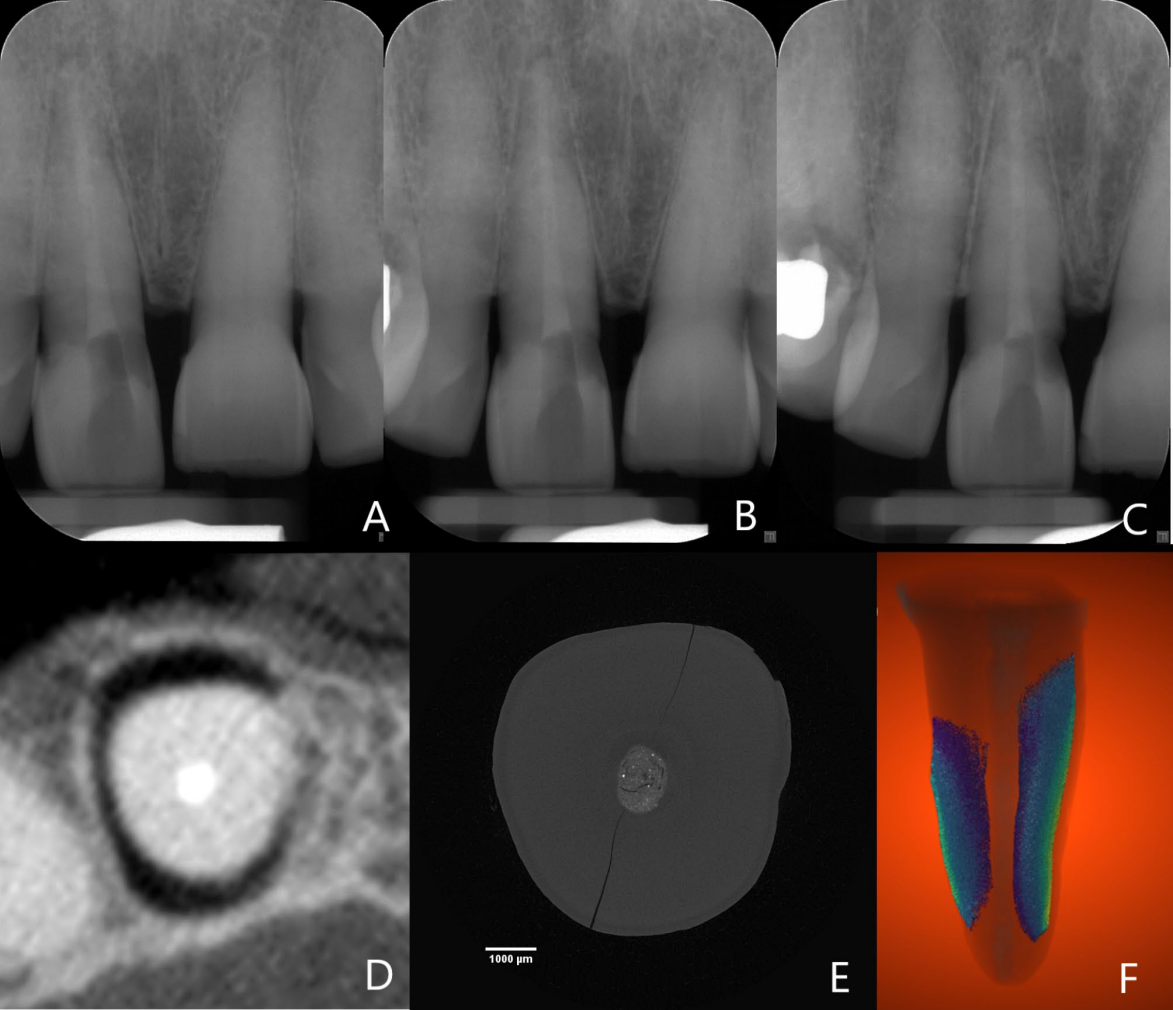
X-ray images of a root-fractured tooth filled with Biodentine™: (**a**) Mesial periapical radiograph. (**b**) Orthogonal periapical radiograph. (**c**) Distal periapical radiograph. (**d**) CBCT ‘child’ axial slice zoomed in on fracture. (**e**) Micro-CT axial slice. (**f**) Three-dimensional micro-CT reconstruction with visualisation of the thickness mesh

X-ray images of a root-fractured tooth filled with Biodentine™ can be appreciated in Fig. 3.

CBCTs were taken for each tooth (Figs. 2c and 3d) with the following predetermined settings: “Child, high resolution: FOV 42 × 50 mm, tube current 8 mA, tube voltage 96 kV, voxel size 100µm, scanning time 12s” and “Adult, high resolution: FOV 50 × 50 mm; tube current 10 mA, tube voltage 96 kV, voxel size 100µm, scanning time 12s”. PRs were taken (Figs. 2d and 3a, b and c) using a dental X-Ray device (Satelec X-Mind^®^, Satelec, France) and a digital phosphor plate system (Vistascan Perio Plus^®^, Dürr Dental Benelux, Belgium) with the following settings: 65 kV, 7 mA, 0.16s. Radiographs were taken in an orthogonal, 10°mesial and 10°distal angulated position with a radiographic holder (Rinn^®^, Dentsply Sirona, USA) with the aiming ring touching the nose of the embalmed head. Thus, a total of 651 PRs (3 per sample) and 434 CBCTs (2 per sample) were taken.

### High-resolution imaging: microcomputed tomography and fracture thickness measurements

Ten out of 42 teeth with VRFs were randomly selected for micro-CT (microcomputed tomography) imaging to assess the VRFs’ dimensions accurately. The teeth were imaged at the custom-built HECTOR scanner of the Ghent University Centre for X-ray Tomography (UGCT) [25]. A tube voltage and a target power of 120 kV and 10 W were used, respectively. A 1 mm thick Al filter was used to reduce beam hardening artefacts. For each sample, 2001 projection images were acquired at an exposure time of 1000ms each, resulting in a total measurement time of almost 40 min. Using geometrical magnification, an isotropic reconstructed voxel size of 9.1 μm was obtained (Fig. 3e). At the used settings of the X-ray source, the resolution was not affected by the focal spot size of the source. Consequently, the raw data were reconstructed to a virtual 3D volume using the in-house developed software package Octopus Reconstruction [26].

The micro-CT images were loaded as an image stack consisting of 16-bit gray-value TIFF images into Dragonfly 4.1 (Object Research Systems, Canada). These images were processed by mean shift filtering and application of an unsharp mask. The region of interest (ROI) for the fractures was manually painted on the image every 30–50 slices and then interpolated. This ROI was used as a mask, and the air was segmented from the fracture zone through thresholding. The air ROI was then refined by isolating the largest 6-connected objects corresponding with the partial root fracture. Finally, a smoothed thickness mesh was created (Fig. 3f).

### Image assessment

Three examiners (a master’s level student (CY), a periodontologist (MD) and an endodontologist (GH) with extended experience in protocolling CBCT images within their speciality) assessed the radiographs. The three observers were calibrated prior to evaluating the images by assessing a series of 20 test images with and without vertical root fractures. After independently scoring the images, the observers discussed any differences in their assessments to reach a consensus on the correct interpretations. This calibration process was repeated after three weeks to reinforce the scoring criteria and ensure consistency among the observers before proceeding with the main study evaluations. All observers were trained in the use of the software. All observations were performed in a dark room on the same Eonis 24” (MDRC-2224 BL) clinical display (Barco™, Belgium) screen with resolution 1920*1200 and front sensor. For each observation session, the images’ order was randomised using a random sequence generator (random.org). Half of the image sample was reassessed by the examiners two weeks or more after the first observations to calculate the intra-examiner agreement.

The PRs were presented as Bitmap Images (BMPs) in a Powerpoint^®^ (Microsoft, USA) presentation. PRs were shown orthogonal only in the first session. All 3 PRs were observed in a separate session: orthogonal, 10° mesial and 10° distal horizontal angulations. The CBCTs were assessed as Digital Imaging and Communications in Medicine (DICOM) files in the Romexis^®^ Viewer software (Planmeca, Finland) with the possibility to adjust image properties such as resolution, contrast, brightness and orientation. The examiners had to score the images as followed: 1 VRF definitely not present, 2 VRF probably not present, 3 unsure, 4 VRF probably present, 5 VRF definitely present according to Gunduz et al., 2013 [27]. These scores were then dichotomised as score 1–3 being a negative and 4–5 being a positive score.

### Statistical analysis

Before sample collection, the authors performed a power analysis in G*Power 3.1.9.2 (Heinrich Heine Universität Düsseldorf, Germany) based on a meta-analysis by Corbella et al., 2014 [16, 28]. At least 36 teeth per group were needed to detect a difference in sensitivity and specificity of 30% with a power of 0.80. The consensus score for the examiners was calculated as the rounded mean of their dichotomised scores, which translates in the most frequent score among the three observers. The authors calculated Gwet’s AC1 intra and inter-examiner agreement using AgreeStat2015.6.1 (©2010 Advanced analytics LLC, USA) [29]. Further data analysis was performed using IBM^®^ SPSS^®^ version 26 (IBM, USA). This analysis included Receiver Operating Characteristic (ROC) analysis and calculation of sensitivity, specificity, positive and negative predictive values, and Diagnostic Odds Ratios (DORs). Paired-sample area difference under the ROC curves under the nonparametric assumption were calculated to compare all radiographic techniques for all samples as well as Biodentine™ and non Biodentine™-filled samples separately. Statistical significance was inferred at *p* < 0.05.

## Results

Inter-rater reliabilities, as well as individual and consensus-score intra-rater reliabilities (Gwet’s AC1) were assessed. These results are detailed in Table 1. Values are graded as poor (< 0.20), fair (0.31–0.40), moderate (0.41–0.60), good (0.61–0.80) or very good (0.81-1.00) [30].

**Table 1:** Intra and Inter-Rater reliability

	Rater 1	Rater 2	Rater 3	Consensus Score Intra-Rater
	1PR			0,81 ± 0,05 (0,72 − 0,90)
Rater 1	1,00 ± 0,00 (1,00–1,00)	0,58 ± 0,05 (0,47 − 0,69)	0,85 ± 0,03 (0,79 − 0,90)	
Rater 2		0,34 ± 0,10 (0,15 − 0,53)	0,52 ± 0,06 (0,40 − 0,64)	
Rater 3			0,73 ± 0,06 (0,61 − 0,85)	
	3PR			0,83 ± 0,04 (0,75 − 0,92)
Rater 1	1,00 ± 0,00 (1,00–1,00)	0,52 ± 0,06 (0,40 − 0,63)	0,76 ± 0,04 (0,68 − 0,84)	
Rater 2		0,17 ± 0,10 (0,00–0,38)	0,28 ± 0,07 (0,14 − 0,43)	
Rater 3			0,63 ± 0,07 (0,49 − 0,78)	
	CBCTchild			0,77 ± 0,05 (0,67 − 0,88)
Rater 1	0,54 ± 0,08 (0,38 − 0,70)	0,36 ± 0,07 (0,22 − 0,50)	0,65 ± 0,05 (0,55 − 0,75)	
Rater 2		0,30 ± 0,10 (0,10 − 0,50)	0,50 ± 0,06 (0,38 − 0,62)	
Rater 3			0,82 ± 0,05 (0,73 − 0,92)	
	CBCTadult			0,86 ± 0,04 (0,79 − 0,94)
Rater 1	0,65 ± 0,07 (0,51 − 0,80)	0,38 ± 0,07 (0,25 − 0,52)	0,55 ± 0,06 (0,43 − 0,66)	
Rater 2		0,63 ± 0,07 (0,50 − 0,78)	0,66 ± 0,05 (0,56 − 0,75)	
Rater 3			0,74 ± 0,06 (0,62 − 0,85)	

Gwet’s AC1 ± SE (95%CI); PR, Periapical Radiograph; CBCT, Cone-Beam Computed Tomography; SE, Standard Error; CI, Confidence Interval

Inter-rater reliability (Gwet’s AC1) between rater 1 and rater 3 ranged from moderate to very good across all imaging types. Reliability between rater 1 and rater 2 was fair for CBCTs and moderate for PRs. Between rater 2 and rater 3, inter-rater reliability varied between fair (for 3PRs), moderate (for 1PR and CBCT with child presetting) and good (for CBCT with adult presetting).

For rater 1, intra-rater reliability (Gwet’s AC1) was very good for the PRs and ranged from moderate to good for the CBCTs. Rater 3 had a good to very good intra-rater reliability. For rater 2, intra-rater reliability was poor for the session with 3PRs and fair for 1PR and the CBCTs with child presetting. It was good for the CBCTs with adult presetting.

The intra-rater score based on the majority-vote consensus varied from good to very good.

There was no significant difference (Pearson’s Chi-squared test) in the number of left (128) or right (89) central incisors between the total amount of observations for all groups (*n* = 217).

The thickness mesh measurements for 10 of the 43 root fractured teeth are available in the supplementary material. The mean fracture thickness ranged from 54.44 μm to 104.39 μm, with standard deviations varying between 10.40 μm and 47.46 μm. Only one tooth had a mean thickness slightly above 100 μm. Two of the ten randomly selected teeth showed outliers with fracture thicknesses slightly exceeding 200 μm.

Table 2 shows the sensitivity, specificity, positive predictive value, negative predictive value, DOR and area under the curve (AUC) with a 95% confidence interval for the consensus scores on all imaging types in the groups without and with Biodentine™ obturation as well as all the samples together.

**Table 2:** Sensitivity, specificity, positive predictive value,negative predictive value, diagnostic odds ratio and area under the curve for consensus observation scores on all imaging types for all samples and the groups without and with Biodentine™

Imaging	Sens	Spec	PPV	NPV	DOR	AUC (95%CI)
All Samples						
1PR	0,09	0,96	0,57	0,62	2,18	0,52 (0,49 − 0,56)
3PR	0,06	0,95	0,42	0,61	1,12	0,5 (0,47 − 0,53)
CBCTchild	0,11	0,85	0,31	0,6	0,66	0,48 (0,43 − 0,52)
CBCTadult	0,06	0,92	0,33	0,6	0,76	0,49 (0,46 − 0,53)
No Biodentine™						
1PR	0,07	0,96	0,43	0,68	1,59	0,51 (0,47 − 0,56)
3PR	0,07	0,96	0,43	0,68	1,59	0,51 (0,47 − 0,56)
CBCTchild	0,16	0,82	0,3	0,67	0,89	0,49 (0,42 − 0,56)
CBCTadult	0,07	0,90	0,25	0,67	0,67	0,48 (0,44 − 0,53)
Biodentine™						
1PR	0,12	0,95	0,71	0,53	2,77	0,54 (0,48 − 0,60)
3PR	0,05	0,93	0,40	0,50	0,67	0,49 (0,44 − 0,54)
CBCTchild	0,05	0,91	0,33	0,49	0,49	0,48 (0,42 − 0,53)
CBCTadult	0,05	0,98	0,67	0,51	2,10	0,51 (0,47 − 0,55)

Sens, Sensitivity; Spec, Specificity; PPV, Positive Predictive Value; NPV, Negative Predictive Value; DOR, Diagnostic Odds Ratio; AUC, Area Under the Curve; CI, Confidence interval; 1PR, 1 Periapical Radiograph; 3PR, 3 Periapical Radiographs; CBCTchild, CBCT with child presetting; CBCTadult, CBCT with adult presetting

Specificity for the detection of VRF’s was consistently higher than sensitivity across all imaging techniques, with specificities ranging from 0.85 to 0.96, indicating a high true negative rate. However, sensitivities were notably low, ranging from 0.05 to 0.16, reflecting a limited ability to correctly identify fractures.

The AUC, which assessed the overall diagnostic accuracy, is scored as poor value starting from 0.6, fair from 0.7, good from 0.8 and excellent from 0.9 [31]. A value under 0.5 means that the model is no better than a random prediction.

The AUC values ranged between 0.48 and 0.54, thus remained below 0.6 for all modalities, suggesting poor performance.

Paired sample area difference under the ROC curves under the nonparametric assumption was performed in between diagnostic imaging techniques for all samples together, as well as separately for the Biodentine™ as non Biodentine™ root-filled samples separately. These results can be found in Table 3.

**Table 3:** *P*-values for paired sample area difference under the ROC curves under the nonparametric assumption in between imaging techniques

Imaging	ΔAUC (95%CI)	ΔSE	*p*-value
All Samples			
1PR*3PR	0,02 (-0,02 − 0,06)	0,19	0,31
1PR*CBCTchild	0,05 (-0,01 − 0,11)	0,20	0,11
1PR*CBCTadult	0,03 (-0,01 − 0,08)	0,19	0,17
3PR*CBCTchild	0,03 (-0,03 − 0,08)	0,20	0,38
3PR*CBCTadult	0,01 (-0,03 − 0,06)	0,30	0,62
CBCTchild*CBCTadult	-0,01 (-0,07 − 0,04)	0,40	0,62
No Biodentine™			
1PR*3PR	0,00 (-0,05 − 0,05)	0,21	1.00
1PR*CBCTchild	0,02 (-0,06 − 0,10)	0,24	0,61
1PR*CBCTadult	0,03 (-0,03 − 0,87)	0,22	0,35
3PR*CBCTchild	0,02 (-0,07 − 0,11)	0,24	0,63
3PR*CBCTadult	0,03 (-0,03 − 0,09)	0,22	0,37
CBCTchild*CBCTadult	0,01 (-0,08 − 0,10)	0,25	0,87
Biodentine™			
1PR*3PR	0,05 (-0,03 − 0,12)	0,24	0,20
1PR*CBCTchild	0,06 (-0,02 − 0,14)	0,24	0,17
1PR*CBCTadult	0,02 (-0,05 − 0,10)	0,23	0,52
3PR*CBCTchild	0,01 (-0,07 − 0,09)	0,23	0,77
3PR*CBCTadult	-0,02 (-0,09 − 0,04)	0,22	0,49
CBCTchild*CBCTadult	-0,04 (-0,10 − 0,03)	0,22	0,32

ΔAUC, Area Under the Curve difference; ΔSE, Standard Error difference; CI, Confidence interval; 1PR, 1 Periapical Radiograph; 3PR, 3 Periapical Radiographs; CBCTchild, CBCT with child presetting; CBCTadult, CBCT with adult presetting

There were no significant differences, all *p*-values were above 0.05. This suggests that none of the imaging modalities provided a statistically significant advantage over the others in detecting VRFs. The DORs ranged from 0 to 2.35, further reflecting the limited diagnostic capability of the imaging techniques used in this study.

## Discussion

This study provides the first comprehensive evaluation of the accuracy of VRF detection in Biodentine™-filled teeth using the Promax 3Dmax CBCT unit, compared to PRs.

The current methodology presents some limitations. The “child settings” for CBCT were intended to simulate clinical scenarios with lower radiation doses for paediatric patients, hypothesizing that a smaller FOV would improve accuracy. However, this potential benefit might be offset by reduced radiation intensity, and the inability to obtain a paediatric embalmed skull, along with similar resolution between CBCT settings, limits direct comparison of their diagnostic accuracy.

The choice of central incisors over premolars and molars, though technically convenient in a complete embalmed skull, might impact generalizability. This can theoretically result in less superposition compared to premolars and molars and thus in higher accuracy rates. However, Biodentine™ is used more often in incisors, for example in root fractures after dental trauma, although such fractures are more often oblique, these can be vertical [3, 4]. Additionally, the sample size, despite performing sample size calculation, may be considered relatively small, limiting the ability to detect smaller relevant differences.

While using a dichotomized mean of a binomial score with multiple observers can simplify analysis, it may lead to a loss of information and statistical power compared to analysing the original 5-point rating scale. Although calculating the mean of binomial scores can summarize data, it might not fully capture observer variability or agreement.

Therefore, to assess intra- and inter-rater reliability, Gwet’s AC-1 was calculated for each observer. Intra-rater reliability was generally sufficient, except for PRs and CBCT with child settings by Rater 2, and CBCT with child settings by Rater 1, where Gwet’s AC1 was below 60, requiring cautious interpretation. Higher intra-rater reliability scores were linked to a high percentage of negative scores. Comparable studies on in-vitro simulated incomplete vertical fractures also showed low reliability or higher reliability but with wide confidence intervals [11, 12, 32]. A systematic review on in vivo accuracy reported variable agreement scores and noted that observer conservativeness could affect sensitivity and specificity [33]. In this study, the known search for root fractures may have led to guessing, contributing to lower inter-rater reliabilities. Most relevant systematic reviews did not mention the levels of agreement [6, 15, 34, 35].

In this study, a consensus score was achieved among the three observers by using the most frequent score. It is important to note that this consensus reflects a higher level of agreement among the observers, despite the noted variability in reliability, particularly with rater 2.

No significant difference between PR, 3PR and CBCT device (with two different device settings) was found for the overall detection of small VRFs in maxillary central incisors in vitro. AUC levels in unfilled teeth did not exceed 0.6, suggesting no significant advantage for CBCT over PR. Brady et al., 2014 found slightly better results with AUC values of 0.540 (0.039 SD) for PR, 0.687 (0.061 SD) for a 3D-Accuitomo (J. Morita, Japan) and 0.659 (0.036 SD) for an i-CAT CBCT device (KaVo, Germany) [11]. CBCT performed significantly better than PR in their study (*p* < 0.05). Guo et al., 2019 did not research the accuracy for PR but found much higher AUC values in all their CBCT devices [36]. Their mean AUC values varied between 0.92 and 0.98, with a value of 0.97 (0.95–0.98 95%CI) for a NewTom VGI unit (NewTom, Italy) with the same voxel size setting as current study (100 μm).

In filled teeth, AUC levels were similarly low, indicating challenges in accurate detection, especially with a high number of false-negative scores. Patel et al., 2013 found even worse accuracies with a more radiopaque root canal filling material (gutta-percha) [12]. Their mean AUC values were 0.501 (0.001 SD) for PR and 0.406 (0.057 SD) for an Accuitomo 3D CBCT scanner (J. Morita, Japan). These values differed significantly (*p* < 0.05).

Current study found no difference in the in vitro accuracy of current CBCT device in detecting small VRFs between non-root-filled and Biodentine™ root-filled maxillary central incisors. In the literature, some evidence suggests affected diagnostic efficacy (mostly lower sensitivities) for VRF detection by radiopaque materials such as gutta-percha and metal posts in the root canal [6, 12, 15, 33, 34, 37–39]. From recent research, it became clear that fibreglass posts (and unfilled teeth) show lower artefact interference than gutta-percha and NiCr posts [40]. Since Biodentine™ has a lower radiopacity than gutta-percha and metal posts, less artefact interference can be suspected [19, 20, 23]. However, due to the overall low accuracies, current study did not permit definitive conclusions. No prior studies specifically explored the impact of Biodentine™ on VRF detection.

It is probable that in vitro studies that showed higher accuracy values induced wider, too wide or too straight VRFs. In this study, specificities ranged from 0.82 to 0.98, while sensitivities were between 0.05 and 0.16. These results, though generally lower than those reported by other in vitro studies [6, 15, 16, 33–35], , align with findings from studies utilizing similar material and methods [11, 12]. The mean VRF thickness in this study was 74.8 μm with a mean SD of 23.9 μm. Most studies that studied smaller fractures, measured thicknesses for thin root fractures between 30 and 170 μm [11–13, 36, 39, 41]. Only one of these studies also used micro-CT to assess the complete volume of the fractures, namely Pinto et al., 2023 used a 40 μm voxel size Nikon XT H 225 industrial computed tomography (CT) scanner in order to produce reference images [39]. Notably, the current study mimics smaller but irregular (clinically relevant) fracture types.

Considering smaller fracture widths, higher spatial resolution is intuitively associated with improved accuracy. Brüllman and Schulze, 2015 reported a theoretically available spatial resolution in a “best possible” experimental scenario of < 3 lp mm^− 1^ with a median value of approximately 2 lp mm^− 1^ [42]. In this case 200 μm spatial resolution would seem optimistic. Even more, the fractures mainly were smaller than 200 μm in width, disregarding the extreme outliers. Higher accuracy of CBCT has been reported in fractures in root-filled teeth with a width > 200 μm or defined as complete root fractures [12, 43]. Two systematic reviews reported higher sensitivity of CBCT when a voxel size < 200 μm or < 250 μm was used [6, 16].

Differences in accuracy between current study and some other in vitro research, could also be caused by the method of tissue and alveolar tissue-mimicking, which can influence image quality. In vitro research is sometimes performed without any tissue-mimicking [44]. Most in vitro research use mandibles layered in wax, sometimes researchers use a foam box filled with water or an acrylic cylinder or sheet or bovine muscle tissue to mimic soft tissues [11, 12, 37, 43, 45, 46]. The current study is unique since all teeth were placed in the maxilla of a dentate Thiel embalmed skull for hard and soft tissue-mimicking. The Thiel embalming technique was first described by W. Thiel, 1999 and was suggested as a supporting technique for surgery and research in the maxillofacial area by Peuker et al., 2001 [24, 47]. This embalming technique gives the possibility to acquire radiographic images with a high diagnostic quality comparable to the real clinical situation without the soft tissues’ rigidity [48]. There was no mimicking of alveolar tissue, as in a clinical situation, buccolingual alveolar bone loss seems to be a common sign in VRFs [49].

The type of CBCT-unit can also influence the image quality and accuracy of VRF detection [45]. One study very recently reported data on the image quality of a comparable but more recent version of the same device [39]. Although a different methodology was used and no accuracy was measured, the authors did report inappropriate subjective image quality compared to reference standards for the detection of small root cracks, even with recently available 75 μm voxel size with and without presence of a gold alloy metallic post. This confirms current findings.

Clinically, these results suggest that neither CBCT nor PRs are reliable standalone diagnostic tools for small VRFs, emphasizing the need for improved imaging techniques or adjunctive diagnostic methods. The study highlights the necessity for clinicians to consider the limitations of current imaging technologies and the potential for misdiagnosis, which could lead to unnecessary treatments or failure to address existing fractures. A recent in vivo study compared PR with CBCT to detect VRFs [50]. The authors found high specificities (0.81 in untreated teeth and 0.89 in root-filled teeth) and low sensitivities for CBCT (0.05 in untreated and 0.33 in root-filled teeth). There was an overall diagnostic accuracy of 0.535 for PR and 0.552 for CBCT. These results were comparable to our findings. Multiple in vivo studies found similar low results in accuracy for CBCT in detection of VRFs, however, it was suggested that accuracy can be enhanced by including clinical signs such as pain on percussion, periodontal pocket formation and mobility [13, 49, 51]. In vivo studies replicate the clinical situation because even minor movements during a CBCT scan can result in motion artifacts, and high-density materials such as crowns and implants in the mouth can also cause artifacts, deteriorating the final image quality [10, 52]. However, in vivo studies are often limited by lack of control groups, technical difficulties in the affirmation method and no information on the actual width of the fractures [53–55]. In vivo research often has the disadvantage of unbalanced prevalence by exclusion of teeth when surgical exploration is unnecessary and inclusion of teeth with suspicion of a VRF without diagnosis on the PR, all of this can affect sensitivity and specificity values [50, 51, 55].

By addressing the hypotheses, this study sheds light on the diagnostic capabilities of PRs and CBCT, particularly in the context of detecting small VRFs in maxillary central incisors. Such insights are crucial for enhancing clinical decision-making, optimizing patient care, and minimizing unnecessary interventions in dental practice.

Thus, our findings underscore the limited sensitivity of PRs for detecting smaller VRFs due to superposition, enlargement, and deformation, consistent with literature [5, 6]. Surprisingly, current CBCT device did not demonstrate superior detection abilities, even in unfilled teeth, aligning with concerns raised regarding image quality [39].

## Conclusion

The null hypotheses were rejected for the current CBCT device (Promax 3Dmax, Planmeca, Finland). There was no significant accuracy improvement of CBCT over periapical radiographs in detecting small vertical root fractures in both non-root-filled and Biodentine™-root-filled maxillary central incisors. A smaller field of view with lower intensity did not enhance detection accuracy.

These results highlight the challenges in accurately detecting small VRFs, emphasizing the need for further research and technological advancements in this domain.

## Electronic supplementary material

Below is the link to the electronic supplementary material.

Supplementary Material 1

Supplementary Material 2

Supplementary Material 3

## Data Availability

All data generated or analysed during this study are included in this published article and its supplementary information files.

## Abbreviations

AUC: Area Under the Curve
BNF: Biodentine™ filled root canal- No Fracture
BPF: Biodentine™ filled root canal- Partial Fracture
CBCT: Cone-Beam Computed Tomography
CI: Confidence Interval
CT: Computed Tomography
DICOM: Digital Imaging and Communications in Medicine
DOR: Diagnostic Odds Ratio
ENF: Empty root canal- No Fracture
EPF: Empty root canal- Partial Fracture
FOV: Field of View
NPV: Negative Predictive Value
PPV: Positive Predictive Value
PR: Periapical Radiograph
ROC: Receiver Operating Characteristic
ROI: Region of Interest
SD: Standard Deviation
SE: Standard Error
VRF: Vertical Root Fracture
