# Supplementary material for: Vertical root fracture detection with cone-beam computed tomography in Biodentine™ filled teeth
Source: BMC Oral Health. 2024 Oct 4;24:1178. doi: 10.1186/s12903-024-04947-7 (PMC11453024; doi:10.1186/s12903-024-04947-7)
Supplement: Supplementary file 3 — Supplementary Material 3 [file 12903_2024_4947_MOESM3_ESM.docx]

Warning # 849 in column 23. Text: en_BE
The LOCALE subcommand of the SET command has an invalid parameter. It could
not be mapped to a valid backend locale.

Data written to C:\Users\...\7 Submitting\5.BMC oral health\3. Minor revision\9 Supplementary material dataset SPSS.xlsx.
73 variables and 217 cases written to range: SPSS.
Variable: Idnr Type: Number Width: 3 Dec: 0
Variable: Toothcontainernr Type: Number Width: 2 Dec: 0
Variable: Group Type: Number Width: 1 Dec: 0
Variable: N1PR_t1_student Type: Number Width: 1 Dec: 0
Variable: N3PR_t1_student Type: Number Width: 1 Dec: 0
Variable: CBCTchild_t1_student Type: Number Width: 1 Dec: 0
Variable: CBCTadult_t1_student Type: Number Width: 1 Dec: 0
Variable: N1PR_t2_student Type: Number Width: 1 Dec: 0
Variable: N3PR_t2_student Type: Number Width: 1 Dec: 0
Variable: CBCTchild_t2_student Type: Number Width: 1 Dec: 0
Variable: CBCTadult_t2_student Type: Number Width: 1 Dec: 0
Variable: N1PR_t1_periodontologist Type: Number Width: 1 Dec: 0
Variable: N3PR_t1_periodontologist Type: Number Width: 1 Dec: 0
Variable: CBCTchild_t1_periodontologist Type: Number Width: 1 Dec: 0
Variable: CBCTadult_t1_periodontologist Type: Number Width: 1 Dec: 0
Variable: N1PR_t2_periodontologist Type: Number Width: 1 Dec: 0
Variable: N3PR_t2_periodontologist Type: Number Width: 1 Dec: 0
Variable: CBCTchild_t2_periodontologist Type: Number Width: 1 Dec: 0
Variable: CBCTadult_t2_periodontologist Type: Number Width: 1 Dec: 0
Variable: N1PR_t1_endodontist Type: Number Width: 1 Dec: 0
Variable: N3PR_t1_endodontist Type: Number Width: 1 Dec: 0
Variable: CBCTchild_t1_endodontist Type: Number Width: 1 Dec: 0
Variable: CBCTadult_t1_endodontist Type: Number Width: 1 Dec: 0
Variable: N1PR_t2_endodontist Type: Number Width: 1 Dec: 0
Variable: N3PR_t2_endodontist Type: Number Width: 1 Dec: 0
Variable: CBCTchild_t2_endodontist Type: Number Width: 1 Dec: 0
Variable: CBCTadult_t2_endodontist Type: Number Width: 1 Dec: 0
Variable: µCTwidth Type: Number Width: 8 Dec: 2
Variable: CBCTmicrot2 Type: Number Width: 8 Dec: 2
Variable: N1PR_t1_studentdich Type: Number Width: 8 Dec: 2
Variable: N3PR_t1_studentdich Type: Number Width: 8 Dec: 2
Variable: CBCTchild_t1_studentdich Type: Number Width: 8 Dec: 2
Variable: CBCTadult_t1_studentdich Type: Number Width: 8 Dec: 2
Variable: N1PR_t2_studentdich Type: Number Width: 8 Dec: 2
Variable: N3PR_t2_studentdich Type: Number Width: 8 Dec: 2
Variable: CBCTchild_t2_studentdich Type: Number Width: 8 Dec: 2
Variable: CBCTadult_t2_studentdich Type: Number Width: 8 Dec: 2
Variable: N1PR_t1_periodontologistdich Type: Number Width: 8 Dec: 2
Variable: N3PR_t1_periodontologistdich Type: Number Width: 8 Dec: 2
Variable: CBCTchild_t1_periodontologistdich Type: Number Width: 8 Dec: 2
Variable: CBCTadult_t1_periodontologistdich Type: Number Width: 8 Dec: 2
Variable: N1PR_t2_periodontologistdich Type: Number Width: 8 Dec: 2
Variable: N3PR_t2_periodontologistdich Type: Number Width: 8 Dec: 2
Variable: CBCTchild_t2_periodontologistdich Type: Number Width: 8 Dec: 2
Variable: CBCTadult_t2_periodontologistdich Type: Number Width: 8 Dec: 2
Variable: N1PR_t1_endodontistdich Type: Number Width: 8 Dec: 2
Variable: N3PR_t1_endodontistdich Type: Number Width: 8 Dec: 2
Variable: CBCTchild_t1_endodontistdich Type: Number Width: 8 Dec: 2
Variable: CBCTadult_t1_endodontistdich Type: Number Width: 8 Dec: 2
Variable: N1PR_t2_endodontistdich Type: Number Width: 8 Dec: 2
Variable: N3PR_t2_endodontistdich Type: Number Width: 8 Dec: 2
Variable: CBCTchild_t2_endodontistdich Type: Number Width: 8 Dec: 2
Variable: CBCTadult_t2_endodontistdich Type: Number Width: 8 Dec: 2
Variable: Overall Type: Number Width: 8 Dec: 2
Variable: Control Type: Number Width: 8 Dec: 2
Variable: Biodentine Type: Number Width: 8 Dec: 2
Variable: Biodornot Type: Number Width: 8 Dec: 2
Variable: N1PR_t1_dich_consensus Type: Number Width: 1 Dec: 0
Variable: N3PR_t1_dich_consensus Type: Number Width: 1 Dec: 0
Variable: CBCTchild_t1_dich_consensus Type: Number Width: 1 Dec: 0
Variable: CBCTadult_t1_dich_consensus Type: Number Width: 1 Dec: 0
Variable: N1PR_t2_dich_consensus Type: Number Width: 1 Dec: 0
Variable: N3PR_t2_dich_consensus Type: Number Width: 1 Dec: 0
Variable: CBCTchild_t2_dich_consensus Type: Number Width: 1 Dec: 0
Variable: CBCTadult_t2_dich_consensus Type: Number Width: 1 Dec: 0
Variable: N1PR_t1_dich_consensus_NB Type: Number Width: 8 Dec: 2
Variable: N3PR_t1_dich_consensus_NB Type: Number Width: 8 Dec: 2
Variable: CBCTchild_t1_dich_consensus_NB Type: Number Width: 8 Dec: 2
Variable: CBCTadult_t1_dich_consensus_NB Type: Number Width: 8 Dec: 2
Variable: N1PR_t1_dich_consensus_B Type: Number Width: 8 Dec: 2
Variable: N3PR_t1_dich_consensus_B Type: Number Width: 8 Dec: 2
Variable: CBCTchild_t1_dich_consensus_B Type: Number Width: 8 Dec: 2
Variable: CBCTadult_t1_dich_consensus_B Type: Number Width: 8 Dec: 2

**File Information**

| **Notes** |  |  |
| --- | --- | --- |
| Output Created |  | 20-SEP-2024 09:17:22 |
| Comments |  |  |
| Input | Data | C:\Users\jwvacker\OneDrive - UGent\Documenten\1 Kliniekhoofd\Research\03_Vertical Tooth fractures\7 Submitting\5.BMC oral health\3. Minor revision\9 Supplementary material dataset SPSS.sav |
|  | Active Dataset | DataSet1 |
|  | Filter | <none> |
|  | Weight | <none> |
|  | Split File | <none> |
|  | N of Rows in Working Data File | 217 |
| Syntax |  | DISPLAY DICTIONARY. |
| Resources | Processor Time | 00:00:00,00 |
|  | Elapsed Time | 00:00:00,00 |

[DataSet1] C:\Users\jwvacker\OneDrive - UGent\Documenten\1 Kliniekhoofd\Research\03_Vertical Tooth fractures\7 Submitting\5.BMC oral health\3. Minor revision\9 Supplementary material dataset SPSS.sav

| **Variable Information** |  |  |  |  |  |  |  |  |
| --- | --- | --- | --- | --- | --- | --- | --- | --- |
| Variable | Position | Label | Measurement Level | Role | Column Width | Alignment | Print Format | Write Format |
| Idnr | 1 | <none> | Scale | Input | 12 | Right | F3 | F3 |
| Toothcontainernr | 2 | <none> | Nominal | Input | 12 | Right | F2 | F2 |
| Group | 3 | <none> | Nominal | Input | 12 | Right | F1 | F1 |
| N1PR_t1_student | 4 | <none> | Nominal | Input | 12 | Right | F1 | F1 |
| N3PR_t1_student | 5 | <none> | Nominal | Input | 12 | Right | F1 | F1 |
| CBCTchild_t1_student | 6 | <none> | Nominal | Input | 12 | Right | F1 | F1 |
| CBCTadult_t1_student | 7 | <none> | Nominal | Input | 12 | Right | F1 | F1 |
| N1PR_t2_student | 8 | <none> | Nominal | Input | 12 | Right | F1 | F1 |
| N3PR_t2_student | 9 | <none> | Nominal | Input | 12 | Right | F1 | F1 |
| CBCTchild_t2_student | 10 | <none> | Nominal | Input | 12 | Right | F1 | F1 |
| CBCTadult_t2_student | 11 | <none> | Nominal | Input | 12 | Right | F1 | F1 |
| N1PR_t1_periodontologist | 12 | <none> | Nominal | Input | 12 | Right | F1 | F1 |
| N3PR_t1_periodontologist | 13 | <none> | Nominal | Input | 12 | Right | F1 | F1 |
| CBCTchild_t1_periodontologist | 14 | <none> | Nominal | Input | 12 | Right | F1 | F1 |
| CBCTadult_t1_periodontologist | 15 | <none> | Nominal | Input | 12 | Right | F1 | F1 |
| N1PR_t2_periodontologist | 16 | <none> | Nominal | Input | 12 | Right | F1 | F1 |
| N3PR_t2_periodontologist | 17 | <none> | Nominal | Input | 12 | Right | F1 | F1 |
| CBCTchild_t2_periodontologist | 18 | <none> | Nominal | Input | 12 | Right | F1 | F1 |
| CBCTadult_t2_periodontologist | 19 | <none> | Nominal | Input | 12 | Right | F1 | F1 |
| N1PR_t1_endodontist | 20 | <none> | Nominal | Input | 12 | Right | F1 | F1 |
| N3PR_t1_endodontist | 21 | <none> | Nominal | Input | 12 | Right | F1 | F1 |
| CBCTchild_t1_endodontist | 22 | <none> | Nominal | Input | 12 | Right | F1 | F1 |
| CBCTadult_t1_endodontist | 23 | <none> | Nominal | Input | 12 | Right | F1 | F1 |
| N1PR_t2_endodontist | 24 | <none> | Nominal | Input | 12 | Right | F1 | F1 |
| N3PR_t2_endodontist | 25 | <none> | Nominal | Input | 12 | Right | F1 | F1 |
| CBCTchild_t2_endodontist | 26 | <none> | Nominal | Input | 12 | Right | F1 | F1 |
| CBCTadult_t2_endodontist | 27 | <none> | Nominal | Input | 12 | Right | F1 | F1 |
| µCTwidth | 28 | <none> | Scale | Input | 12 | Right | F8.2 | F8.2 |
| CBCTmicrot2 | 29 | <none> | Scale | Input | 12 | Right | F8.2 | F8.2 |
| N1PR_t1_studentdich | 30 | N1PR_t1_studentdich | Nominal | Input | 21 | Right | F8.2 | F8.2 |
| N3PR_t1_studentdich | 31 | N3PR_t1_studentdich | Nominal | Input | 21 | Right | F8.2 | F8.2 |
| CBCTchild_t1_studentdich | 32 | CBCTchild_t1_studentdich | Nominal | Input | 26 | Right | F8.2 | F8.2 |
| CBCTadult_t1_studentdich | 33 | CBCTadult_t1_studentdich | Nominal | Input | 26 | Right | F8.2 | F8.2 |
| N1PR_t2_studentdich | 34 | N1PR_t2_studentdich | Nominal | Input | 21 | Right | F8.2 | F8.2 |
| N3PR_t2_studentdich | 35 | N3PR_t2_studentdich | Nominal | Input | 21 | Right | F8.2 | F8.2 |
| CBCTchild_t2_studentdich | 36 | CBCTchild_t2_studentdich | Nominal | Input | 26 | Right | F8.2 | F8.2 |
| CBCTadult_t2_studentdich | 37 | CBCTadult_t2_studentdich | Nominal | Input | 26 | Right | F8.2 | F8.2 |
| N1PR_t1_periodontologistdich | 38 | N1PR_t1_periodontologistdich | Nominal | Input | 30 | Right | F8.2 | F8.2 |
| N3PR_t1_periodontologistdich | 39 | N3PR_t1_periodontologistdich | Nominal | Input | 30 | Right | F8.2 | F8.2 |
| CBCTchild_t1_periodontologistdich | 40 | CBCTchild_t1_periodontologistdich | Nominal | Input | 34 | Right | F8.2 | F8.2 |
| CBCTadult_t1_periodontologistdich | 41 | CBCTadult_t1_periodontologistdich | Nominal | Input | 34 | Right | F8.2 | F8.2 |
| N1PR_t2_periodontologistdich | 42 | N1PR_t2_periodontologistdich | Nominal | Input | 30 | Right | F8.2 | F8.2 |
| N3PR_t2_periodontologistdich | 43 | N3PR_t2_periodontologistdich | Nominal | Input | 30 | Right | F8.2 | F8.2 |
| CBCTchild_t2_periodontologistdich | 44 | CBCTchild_t2_periodontologistdic | Nominal | Input | 34 | Right | F8.2 | F8.2 |
| CBCTadult_t2_periodontologistdich | 45 | CBCTadult_t2_periodontologistdich | Nominal | Input | 34 | Right | F8.2 | F8.2 |
| N1PR_t1_endodontistdich | 46 | N1PR_t1_endodontistdich | Nominal | Input | 25 | Right | F8.2 | F8.2 |
| N3PR_t1_endodontistdich | 47 | N3PR_t1_endodontistdich | Nominal | Input | 25 | Right | F8.2 | F8.2 |
| CBCTchild_t1_endodontistdich | 48 | CBCTchild_t1_endodontistdich | Nominal | Input | 30 | Right | F8.2 | F8.2 |
| CBCTadult_t1_endodontistdich | 49 | CBCTadult_t1_endodontistdich | Nominal | Input | 30 | Right | F8.2 | F8.2 |
| N1PR_t2_endodontistdich | 50 | N1PR_t2_endodontistdich | Nominal | Input | 25 | Right | F8.2 | F8.2 |
| N3PR_t2_endodontistdich | 51 | N3PR_t2_endodontistdich | Nominal | Input | 25 | Right | F8.2 | F8.2 |
| CBCTchild_t2_endodontistdich | 52 | CBCTchild_t2_endodontistdich | Nominal | Input | 30 | Right | F8.2 | F8.2 |
| CBCTadult_t2_endodontistdich | 53 | CBCTadult_t2_endodontist | Nominal | Input | 30 | Right | F8.2 | F8.2 |
| Overall | 54 | Overall | Nominal | Input | 10 | Right | F8.2 | F8.2 |
| Control | 55 | Control | Nominal | Input | 10 | Right | F8.2 | F8.2 |
| Biodentine | 56 | Biod | Nominal | Input | 12 | Right | F8.2 | F8.2 |
| Biodornot | 57 | Biodentine or not | Nominal | Input | 11 | Right | F8.2 | F8.2 |
| N1PR_t1_dich_consensus | 58 | <none> | Nominal | Input | 24 | Right | F1 | F1 |
| N3PR_t1_dich_consensus | 59 | <none> | Nominal | Input | 24 | Right | F1 | F1 |
| CBCTchild_t1_dich_consensus | 60 | <none> | Nominal | Input | 29 | Right | F1 | F1 |
| CBCTadult_t1_dich_consensus | 61 | <none> | Nominal | Input | 29 | Right | F1 | F1 |
| N1PR_t2_dich_consensus | 62 | <none> | Nominal | Input | 24 | Right | F1 | F1 |
| N3PR_t2_dich_consensus | 63 | <none> | Nominal | Input | 24 | Right | F1 | F1 |
| CBCTchild_t2_dich_consensus | 64 | <none> | Nominal | Input | 29 | Right | F1 | F1 |
| CBCTadult_t2_dich_consensus | 65 | <none> | Nominal | Input | 29 | Right | F1 | F1 |
| N1PR_t1_dich_consensus_NB | 66 | <none> | Nominal | Input | 27 | Right | F8.2 | F8.2 |
| N3PR_t1_dich_consensus_NB | 67 | <none> | Nominal | Input | 27 | Right | F8.2 | F8.2 |
| CBCTchild_t1_dich_consensus_NB | 68 | <none> | Nominal | Input | 32 | Right | F8.2 | F8.2 |
| CBCTadult_t1_dich_consensus_NB | 69 | <none> | Nominal | Input | 32 | Right | F8.2 | F8.2 |
| N1PR_t1_dich_consensus_B | 70 | <none> | Nominal | Input | 26 | Right | F8.2 | F8.2 |
| N3PR_t1_dich_consensus_B | 71 | <none> | Nominal | Input | 26 | Right | F8.2 | F8.2 |
| CBCTchild_t1_dich_consensus_B | 72 | <none> | Nominal | Input | 31 | Right | F8.2 | F8.2 |
| CBCTadult_t1_dich_consensus_B | 73 | <none> | Nominal | Input | 31 | Right | F8.2 | F8.2 |

| Variables in the working file |  |  |  |  |  |  |  |  |
| --- | --- | --- | --- | --- | --- | --- | --- | --- |

| **Variable Values** |  |  |
| --- | --- | --- |
| Value |  | Label |
| Group | 1 | ENF |
|  | 2 | EPF |
|  | 3 | BNF |
|  | 4 | BPF |
| N1PR_t1_student | 1 | VRFdefinitely not present |
|  | 2 | VRF probably not present |
|  | 3 | unsure |
|  | 4 | VRF probably present |
|  | 5 | VRF definitely present |
| N3PR_t1_student | 1 | VRFdefinitely not present |
|  | 2 | VRF probably not present |
|  | 3 | unsure |
|  | 4 | VRF probably present |
|  | 5 | VRF definitely present |
| CBCTchild_t1_student | 1 | VRFdefinitely not present |
|  | 2 | VRF probably not present |
|  | 3 | unsure |
|  | 4 | VRF probably present |
|  | 5 | VRF definitely present |
| CBCTadult_t1_student | 1 | VRFdefinitely not present |
|  | 2 | VRF probably not present |
|  | 3 | unsure |
|  | 4 | VRF probably present |
|  | 5 | VRF definitely present |
| N1PR_t2_student | 1 | VRFdefinitely not present |
|  | 2 | VRF probably not present |
|  | 3 | unsure |
|  | 4 | VRF probably present |
|  | 5 | VRF definitely present |
| N3PR_t2_student | 1 | VRFdefinitely not present |
|  | 2 | VRF probably not present |
|  | 3 | unsure |
|  | 4 | VRF probably present |
|  | 5 | VRF definitely present |
| CBCTchild_t2_student | 1 | VRFdefinitely not present |
|  | 2 | VRF probably not present |
|  | 3 | unsure |
|  | 4 | VRF probably present |
|  | 5 | VRF definitely present |
| CBCTadult_t2_student | 1 | VRFdefinitely not present |
|  | 2 | VRF probably not present |
|  | 3 | unsure |
|  | 4 | VRF probably present |
|  | 5 | VRF definitely present |
| N1PR_t1_periodontologist | 1 | VRFdefinitely not present |
|  | 2 | VRF probably not present |
|  | 3 | unsure |
|  | 4 | VRF probably present |
|  | 5 | VRF definitely present |
| N3PR_t1_periodontologist | 1 | VRFdefinitely not present |
|  | 2 | VRF probably not present |
|  | 3 | unsure |
|  | 4 | VRF probably present |
|  | 5 | VRF definitely present |
| CBCTchild_t1_periodontologist | 1 | VRFdefinitely not present |
|  | 2 | VRF probably not present |
|  | 3 | unsure |
|  | 4 | VRF probably present |
|  | 5 | VRF definitely present |
| CBCTadult_t1_periodontologist | 1 | VRFdefinitely not present |
|  | 2 | VRF probably not present |
|  | 3 | unsure |
|  | 4 | VRF probably present |
|  | 5 | VRF definitely present |
| N1PR_t2_periodontologist | 1 | VRFdefinitely not present |
|  | 2 | VRF probably not present |
|  | 3 | unsure |
|  | 4 | VRF probably present |
|  | 5 | VRF definitely present |
| N3PR_t2_periodontologist | 1 | VRFdefinitely not present |
|  | 2 | VRF probably not present |
|  | 3 | unsure |
|  | 4 | VRF probably present |
|  | 5 | VRF definitely present |
| CBCTchild_t2_periodontologist | 1 | VRFdefinitely not present |
|  | 2 | VRF probably not present |
|  | 3 | unsure |
|  | 4 | VRF probably present |
|  | 5 | VRF definitely present |
| CBCTadult_t2_periodontologist | 1 | VRFdefinitely not present |
|  | 2 | VRF probably not present |
|  | 3 | unsure |
|  | 4 | VRF probably present |
|  | 5 | VRF definitely present |
| N1PR_t1_endodontist | 1 | VRFdefinitely not present |
|  | 2 | VRF probably not present |
|  | 3 | unsure |
|  | 4 | VRF probably present |
|  | 5 | VRF definitely present |
| N3PR_t1_endodontist | 1 | VRFdefinitely not present |
|  | 2 | VRF probably not present |
|  | 3 | unsure |
|  | 4 | VRF probably present |
|  | 5 | VRF definitely present |
| CBCTchild_t1_endodontist | 1 | VRFdefinitely not present |
|  | 2 | VRF probably not present |
|  | 3 | unsure |
|  | 4 | VRF probably present |
|  | 5 | VRF definitely present |
| CBCTadult_t1_endodontist | 1 | VRFdefinitely not present |
|  | 2 | VRF probably not present |
|  | 3 | unsure |
|  | 4 | VRF probably present |
|  | 5 | VRF definitely present |
| N1PR_t2_endodontist | 1 | VRFdefinitely not present |
|  | 2 | VRF probably not present |
|  | 3 | unsure |
|  | 4 | VRF probably present |
|  | 5 | VRF definitely present |
| N3PR_t2_endodontist | 1 | VRFdefinitely not present |
|  | 2 | VRF probably not present |
|  | 3 | unsure |
|  | 4 | VRF probably present |
|  | 5 | VRF definitely present |
| CBCTchild_t2_endodontist | 1 | VRFdefinitely not present |
|  | 2 | VRF probably not present |
|  | 3 | unsure |
|  | 4 | VRF probably present |
|  | 5 | VRF definitely present |
| CBCTadult_t2_endodontist | 1 | VRFdefinitely not present |
|  | 2 | VRF probably not present |
|  | 3 | unsure |
|  | 4 | VRF probably present |
|  | 5 | VRF definitely present |
| N1PR_t1_studentdich | .00 | NF |
|  | 1.00 | F |
| N3PR_t1_studentdich | .00 | NF |
|  | 1.00 | F |
| CBCTchild_t1_studentdich | .00 | NF |
|  | 1.00 | F |
| CBCTadult_t1_studentdich | .00 | NF |
|  | 1.00 | F |
| N1PR_t2_studentdich | .00 | NF |
|  | 1.00 | F |
| N3PR_t2_studentdich | .00 | NF |
|  | 1.00 | F |
| CBCTchild_t2_studentdich | .00 | NF |
|  | 1.00 | F |
| CBCTadult_t2_studentdich | .00 | NF |
|  | 1.00 | F |
| N1PR_t1_periodontologistdich | .00 | NF |
|  | 1.00 | F |
| N3PR_t1_periodontologistdich | .00 | NF |
|  | 1.00 | F |
| CBCTchild_t1_periodontologistdich | .00 | NF |
|  | 1.00 | F |
| CBCTadult_t1_periodontologistdich | .00 | NF |
|  | 1.00 | F |
| N1PR_t2_periodontologistdich | .00 | NF |
|  | 1.00 | F |
| N3PR_t2_periodontologistdich | .00 | NF |
|  | 1.00 | F |
| CBCTchild_t2_periodontologistdich | .00 | NF |
|  | 1.00 | F |
| CBCTadult_t2_periodontologistdich | .00 | NF |
|  | 1.00 | F |
| N1PR_t1_endodontistdich | .00 | NF |
|  | 1.00 | F |
| N3PR_t1_endodontistdich | .00 | NF |
|  | 1.00 | F |
| CBCTchild_t1_endodontistdich | .00 | NF |
|  | 1.00 | F |
| CBCTadult_t1_endodontistdich | .00 | NF |
|  | 1.00 | F |
| N1PR_t2_endodontistdich | .00 | NF |
|  | 1.00 | F |
| N3PR_t2_endodontistdich | .00 | NF |
|  | 1.00 | F |
| CBCTchild_t2_endodontistdich | .00 | NF |
|  | 1.00 | F |
| CBCTadult_t2_endodontistdich | .00 | NF |
|  | 1.00 | F |
| Overall | .00 | NF |
|  | 1.00 | F |
| Control | .00 | NF |
|  | 1.00 | F |
| Biodentine | .00 | NF |
|  | 1.00 | F |
| Biodornot | .00 | NB |
|  | 1.00 | B |
